# OpenKnowledge for peer-to-peer experimentation in protein identification by MS/MS

**DOI:** 10.1186/1759-4499-3-3

**Published:** 2011-12-22

**Authors:** Siu-wai Leung, Xueping Quan, Paolo Besana, Qian Li, Mark Collins, Dietlind Gerloff, Dave Robertson

**Affiliations:** 1 School of Informatics, University of Edinburgh, Edinburgh EH8 9AB, UK; 2 State Key Laboratory of Quality Research in Chinese Medicine, University of Macau, Macao SAR, China; 3 Institute of Chinese Medical Sciences, University of Macau, Macao SAR, China; 4 Division of Ecology and Evolution, Imperial College London, London SW7 2AZ, UK; 5 Department of Biomolecular Engineering, University of California, Santa Cruz, CA 95064, USA

## Abstract

**Background:**

Traditional scientific workflow platforms usually run individual experiments with little evaluation and analysis of performance as required by automated experimentation in which scientists are being allowed to access numerous applicable workflows rather than being committed to a single one. Experimental protocols and data under a peer-to-peer environment could potentially be shared freely without any single point of authority to dictate how experiments should be run. In such environment it is necessary to have mechanisms by which each individual scientist (peer) can assess, locally, how he or she wants to be involved with others in experiments. This study aims to implement and demonstrate simple peer ranking under the OpenKnowledge peer-to-peer infrastructure by both simulated and real-world bioinformatics experiments involving multi-agent interactions.

**Methods:**

A simulated experiment environment with a peer ranking capability was specified by the Lightweight Coordination Calculus (LCC) and automatically executed under the OpenKnowledge infrastructure. The peers such as MS/MS protein identification services (including web-enabled and independent programs) were made accessible as OpenKnowledge Components (OKCs) for automated execution as peers in the experiments. The performance of the peers in these automated experiments was monitored and evaluated by simple peer ranking algorithms.

**Results:**

Peer ranking experiments with simulated peers exhibited characteristic behaviours, e.g., power law effect (a few dominant peers dominate), similar to that observed in the traditional Web. Real-world experiments were run using an interaction model in LCC involving two different types of MS/MS protein identification peers, *viz*., peptide fragment fingerprinting (PFF) and *de novo *sequencing with another peer ranking algorithm simply based on counting the successful and failed runs. This study demonstrated a novel integration and useful evaluation of specific proteomic peers and found MASCOT to be a dominant peer as judged by peer ranking.

**Conclusion:**

The simulated and real-world experiments in the present study demonstrated that the OpenKnowledge infrastructure with peer ranking capability can serve as an evaluative environment for automated experimentation.

## Background

This study demonstrates the use of peer ranking of multi-agents (peers) on a peer-to-peer scientific experimentation platform. Experiments with simulated peers were conducted to show that sophisticated peer ranking algorithms are applicable to the OpenKnowledge infrastructure. Experimental demonstration of peer ranking, albeit with a simple algorithm, of actual computational services for tandem mass spectrometry (MS/MS) protein identification were still useful in monitoring the performance of peers during their interactions. The rest of this background section will describe the concepts and technologies applicable to this study.

### OpenKnowledge infrastructure

**Figure 1:**
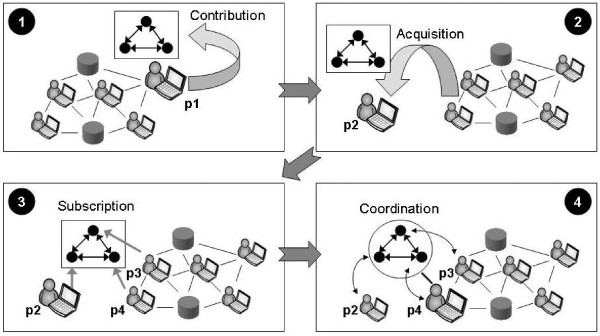
**Sharing experimental protocols in OpenKnowledge system**.

It is commonplace for bioinformaticians to use experimental protocols that coordinate programs with the aim of finding novel results. It is uncommon, however, for these protocols to be viewed as a key aspect of experiment design. Instead, the protocols normally are viewed as part of the experimental infrastructure; they specify how to run the experiment but do not directly define the experiment. For this reason, the protocols are seldom made sharable even if they are reusable. The OpenKnowledge system provides a method for describing experimental protocols in a high level declarative language called Lightweight Coordination Calculus (LCC) so that the specification of a protocol can be independent of the means used to automate it and a general-purpose mechanism (the OpenKnowledge system) through which these protocols may be shared within peer groups (Figure 1).

In Figure 1, frame 1 (top left) depicts the peer to peer network of computers, some being operated by humans others being automated programs (such as database services). Protocols are designed by humans and contributed to the peer to peer network by an advertisement mechanism (using tags to give a simple keyword-based characterisation of the protocol). In frame 1, peer p1 contributes an experimental protocol with three interacting roles (depicted as black circles in the diagram). Other peers acquire experimental protocols by posting a query to the peer network and receiving a list of protocols with (approximately) matching tags. This is analogous to searching for a Web page except that routing through the network replaces a centralised system of query servers. In frame 2, peer p2, acquires the experimental protocol contributed by p1. If a peer finds an experiment that is useful to it then it may offer it for subscription by appropriate collaborating peers. If the choice of peers is already determined (e.g. when the experiment involves coordinating a known collection of Web services) then there is no need for subscription by peers other than the one initiating the protocol. If, however, the initiator does not know which peers might undertake some of the roles in the experimental protocol then it may offer it for subscription (and in turn it will be offered subscribers as these become available). In frame 3, peer p2 offers the experimental protocol for subscription and peers p3 and p4 subscribe to appropriate roles. Once a protocol is fully subscribed it can be used (*via *the OpenKnowledge interpreter) to coordinate the interaction between subscribed peers. This can be done by maintaining the coordination on a server (the traditional method); or by distributing the roles to the peers (a "pure" peer to peer method); or by selecting one of the peer group to act as host. The latter method is used in the current OpenKnowledge system but the experimental protocol is defined independently of this choice (so the protocols don't have to change if the infrastructure for coordination changes). In frame 4, peer p4 is hosting the coordination between p2, p3 and itself.

Crucial to this view of experimentation (Figure 1) is the idea that a single, formal specification describes the abstract experiment design and is also capable of being automated in a simple, uniform way to perform actual experiments. This basic idea is not new-the idea of executable specifications (explained in the context of LCC in [1]) permeates declarative programming (giving a basis for logic programming and functional programming) while in bioinformatics there exist systems such as Taverna [2] that provide design tools for scientific workflow specifications and, in the MyExperiment [3] system, allow these to be tagged with meta-data (analogous to frame 1 in Figure 1) while also supplying infrastructure for executing these workflows (analogous to frame 4 in Figure 1). Where OpenKnowledge differs from Taverna (and similar bioinformatics workflow and pipeline systems) is that we generalise these principles to a peer to peer setting and for generic (not only bioinformatics) applications such as emergency response (see the OpenKnowledge Web site at http://www.openk.org for example applications and scenarios). Specifically:

OpenKnowledge is not specifically targeted at workflow. Although it can be used to implement workflows, its core language (LCC, described later) is a generic, declarative language for describing coordination between processes that synchronise through message passing. In later sections of this paper we show practical bioinformatics experiments that require the facilities of this sort of declarative programming language.

OpenKnowledge is targeted at sharing of detailed experimental protocols. By analogy to the current practice of writing scripts for combining programs in bioinformatics experiments, we assume that a large scale system for sharing experimental protocols would have to deal with large numbers of quite specific protocols, often designed independently by peers. Effective methods of finding, reviewing and repeating experimental protocols then become an important component of the basic science because without such methods it would be unclear what science had been done.

When conducting experimental studies (or amassing information to support experimental studies) from Internet sources, each scientist (or group) may adopt a variety of roles as information providers, consumers or modifiers. Often these roles are narrowly specific, as for example the role one adopts when canvassing trusted sources for information about specific proteins and applying assessment metrics to these that are appropriate to a particular style of experimentation. In science, the roles we adopt and the specific ways in which we discharge the obligations of those roles are fundamental to establishing peer groups of "like minded" scientists in pursuit of related goals by compatible means. The need to be precise about such obligations is strongly felt in traditional science-hence the use of rigid conventions for description of experimental method and monitoring of its execution *via *laboratory notebooks, enabling experiments to be monitored, replicated and re-used. Analogous structure is beginning to emerge in Internet based science. For example the structure of Web service composition in Taverna provides a record of the associations between services when using these to manipulate scientific data. Like Taverna, we describe interactions. Unlike Taverna, our interaction models are part of a system for peer-to-peer communication in which specifications of complementary roles in experimentation are shared between peers as a means of communicating and coordinating experiments.

### The lightweight coordination calculus (LCC)

An interaction model in LCC is a set of clauses each of the form R :: D, where R denotes the role in the interaction and D is the definition of the role. Roles are of the form a(T, P), where T gives the type of role and P is an identifier for the individual peer undertaking that role. The definition of performance of a role is constructed using combinations of the sequence operator (then) or choice operator (or) to connect messages and changes of role. Messages are either outgoing to another peer in a given role (= >) or incoming from another peer in a given role (< =). Message input/output or change of role can be governed by constraints (connected by the "←" operator) which may be conjunctive or disjunctive. Constraints can be satisfied *via *shared components registered with http://www.openk.org, so that complex (possibly interactive) solving methods can be shared along with interaction models; or they can be calls to services with private data and reasoning methods. Variables begin with upper case characters.

**Figure 2:**
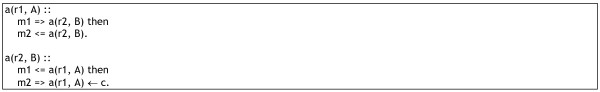
**A basic interaction model in LCC**.

Although it is not shown in the example of Figure 2, role definitions in LCC can be recursive and the language supports structured terms in addition to variables and constants so that, although its syntax is simple, it can represent sophisticated interactions. Notice also that role definitions are "stand alone" in the sense that each role definition specifies all the information needed to complete that role. This means that definitions for roles can be distributed across a network of computers and (assuming the LCC definition is well engineered) will synchronise through message passing while otherwise operating independently. Mathching of output messages from one peer to input messages of another is achieved by simple pattern matching, since (although operating independently) the roles were originally defined to work together. More sophisticated forms of input/output matching have been defined for LCC (to allow for more sophisticated ontology matching) but these are not the subject of this paper. For a more detailed introduction to LCC, see [1].

The model in Figure 2 defines an interaction between peers in two roles, r1 and r2. In the interaction some peer, A, in role r1, would send the message, m1, to peer B in role r2; then it would wait for a reply from peer B with message m2. Conversely, peer, B, in role r2, would wait for the message, m1, from peer A in role r1; then it would send a reply to peer A with message m2 if it can satisfy the constraint c.

### Protein Identifications by MS/MS

Tandem mass spectrometry (MS/MS) is a technique to measure the mass of sample, and has been used for protein sequence analysis for more than two decades [4]. A number of informatics algorithms have been developed to identify protein sequences from the MS spectra with various degrees of accuracy for different types of proteins [5-7]. The basic idea to correlate the spectrum data with theoretical sequences derived from known sequences and their spectra is complicated by the events happened before, during, and after protein synthesis in the cells, i.e., pre-, co-, and post-translational modifications. As such, MS/MS protein identification techniques have some specialisation for handling specific issues. For instance, some techniques are better to reduce complexity of MS spectra and others are better handle modifications or mutations.

The two principal approaches for protein identifications by MS/MS include peptide fragment fingerprinting (PFF) and *de novo *sequencing. PFF algorithms, is performed specifically for candidate peptides extracted from a database by building theoretical model spectra from theoretical peptides and measuring the similarity between the experimental spectra and the modelled ones. Most of the search engines, including MASCOT [5], OMSSA [6], SEQUEST [7], are available both as standalone programs enquiring local copy of public genomic-translated databases (GTDB), or as web services connected to online GTDBs. The main drawback of this approach is that it can only be used in situations where the genome has been sequenced and all predicted proteins for the genome are known. This approach is not suitable for the proteins with missing post-translational modifications (PTMs) and from unsequenced genomes.

The *de novo *sequencing approach infers knowledge about the peptide sequence independently of any information extracted from a pre-existing protein or DNA database [8]. Then, the inferred complete or partial sequences are compared to theoretical sequences according to sequence similarity. It is noted that *de novo *can take a pseudo PFF approach to building a pseudo sequence database on the fly: the sequences are generated by determining all possible amino acid compositions with a total mass matching the experimental precursor mass, and then, for each composition, by determining all possible amino acid permutations. Subsequently, theoretical spectra are computed from the pseudo sequences and common peaks between experimental and theoretical spectrum are counted to find the best matched sequences. Some information, e.g., more accurate precursor masses and range in the number of amino acids, would help reduce the combinational complexity.

For improving the accuracy of the MS/MS protein identification, combining knowledge of applicable methods is plausible [9]. However, this idea of combining methods is yet to take on seriously, let alone to implement and validate. And due to the lack of peer performance monitoring in current experimentation platforms, it would be difficult to evaluate plausible combinations of methods. Thus, a peer ranking facility was implemented and experimented under the peer-to-peer OpenKnowledge infrastructure for performance monitoring. With the peer ranking facility, we also test the feasibility of a simple interaction model that combines both PFF and *de novo *methods of MS/MS protein identification.

### Experimentation with peer ranking

Our peer ranking algorithm for monitoring the simulated peers follows the style of the PageRank algorithm [10]. It works by assigning a ranking to a peer at any given time as a function of its previous ranking modified by the rankings of each peer with which it has interacted (we refer below to such peers as supporting peers). The modification made by each of these peers is spread equally across the set of peers which it supports. In the algorithm, we assume that we have available minimal information about interactions-only knowing for each interaction which peers were involved in it and whether it completed successfully. This information is less than that we can obtain routinely (since the coordinating peer has access to all the structure of a completed interaction) but we make this simplification to make the implementation less domain-dependent and to explore how far we can go with the bare minimum of data. For proteomic experiments, we implemented an even simpler algorithm which should be adequate to demonstrate that certain peers are dominant after a period of interactions in real-world experiments.

This study aims to test peer ranking under OpenKnowledge infrastructure using simulated experiments and real-world bioinformatics experiments. The simulated experiments involved simulated peers assigned with different roles for interactions. The real-world bioinformatics experiments were conducted with multiple peers performing different methods of peptide fragment fingerprinting (PFF) and *de novo *sequencing under OpenKnowledge infrastructure, in which the interaction among these peers was coordinated according to an interaction model.

## Methods

### Peer ranking algorithm

The peer ranking algorithm used for monitoring simulated peers in this study was based on PageRank [10], which notations and formulation were specified and implemented as follows:

Given:

A data set of initial rankings for each peer, each of the form cr(P, T) = R

A data set of records of successful or failed interactions, each of the form im(T, I, P_I_, CP)

where:

P is the identifier of a peer

T is either "pos" (denoting the positive ranking) or "neg" (denoting the negative ranking)

R is the numerical magnitude of the (positive or negative) ranking, R_p _is positive ranking, R_n _is negative ranking

I is the identifier of the interaction model

P_i _is the initiating peer for an interaction

CP is the set of peers that subscribe to the interaction initiated by P_i_

The algorithm for calculating the current rank of a peer was then as follows (where i is the empirically chosen number of iterations used to obtain stable ranking values:

◦ For i iterations:

◦ For each peer, P:

▪ Calculate rank(P) = (R_p_, R_n_)

▪ assign cr(P, pos) = R_p_

▪ assign cr(P, neg) = R_n_

rank(P) calculates the current rank for peer P, where d is an empirically chosen damping value used to tune the ranking system (a frequently used value in page ranking is 0.85):

$$\text{rank}\left(\text{P}\right)=\left(\left(1-\text{d}\right)+\text{d}*\text{r}\left(\text{s}\left(\text{pos},\text{P}\right),\text{pos}\right),\left(1-\text{d}\right)+\text{d}*\text{r}\left(\text{s}\left(\text{neg},\text{P}\right),\text{neg}\right)\right)$$

s(T, P) gives the list of peers supporting peer P. If T is "pos" then this is the set of positive support or if T is "neg" this is the set of negative support. Note that this is a list (which may contain duplicates) rather than a set because we are interested in the number of times each peer is supported.

$$\text{s}\left(\text{T},\text{P}\right)=\left[\text{Ps}|\text{a}\left(\text{P},\text{T},\text{Ps}\right)\right]$$

a(P, T, Ps) is true when peer P is supported by peer Ps either positively (if T is "pos") or negatively (if T is "neg"). Note that this may (intentionally) generate the same instance of Ps more than once. This allows us to count the number of times the same peer is supported.

$$\text{a}\left(\text{P},\text{T},\text{Ps}\right)\;\text{if}\;\text{im}\left(\text{T},\text{I},{\text{P}}_{\text{i}},\text{CP}\right)\;\text{and}\;\left(\left(\text{P}={\text{P}}_{\text{i}}\;\text{and}\;\text{Ps}\in \text{CP}\right)\;\text{or}\;\left(\text{P}\in \text{CP}\;\text{and}\;\text{Ps}={\text{P}}_{\text{I}}\right)\right)$$

r(S, T) is the sum of the current ranks for all the peers in peer set, S, each peer's rank being divided by the number of peers it supports (to apportion the influence of the rank evenly across those supported peers). If T is "pos" then this is the sum of ranks from positive associations or if T is "neg" this is the sum of ranks from negative associations. In other words, r(S, T) is the sum for all P in peer set S of cr(P, T)/L, where L is the number of peers supported by P.

### Experiments on simulated peers

**Figure 3:**
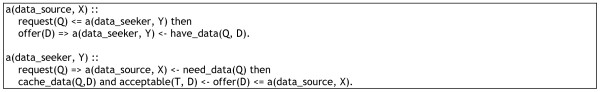
**An interaction model for competitive data sharing**.

The experiments with simulated peers used basic interaction model for data sharing as specified in Figure 3. The interaction model described two roles: a data source which offers a data set to a data seeker if it receives a request for data from the seeker, and a data seeker which requests data for a query and caches the data received (testing also that the data set was of acceptable overall quality). In the interaction model as depicted in Figure 3, X is the identifier for the data source; Y is the identifier for the data seeker; have_data(Q, D) generates the best available set of data, D, known to X for query, Q; need_data(Q) generates a data query, Q for Y; cache_data(Q, D) merges the data set, D with the data cached by Y for query, Q; and acceptable(T, D) succeeds when the data set, D, has a mean quality level that exceeds the threshold T (note that T is set to a specific value).

This interaction model had no representation of the provenance of the data but there was a point in the interaction, when the data seeker sent its request to a data source, when an appropriate data source needed to be chosen. At this point the data seeker could, if it so chose, use peer rank information to select the highest ranking peer. The rank of a data source peer in this interaction, in turn, depended on how frequently the data seekers with which it interacted found the quality of the data it supplied to be acceptable (otherwise the *acceptable *constraint in the data seeker role failed and consequently the interaction overall failed). This gave a feedback loop from supply to ranking *via *the peers that requested data.

To make our simulation as simple as possible we represented data quality as a number ranging from 0 (lowest quality) to 1 (top quality). Instead of storing actual data elements, the peers in our simulation stored these quality values (representing the quality of a data item). Although in the OpenKnowledge framework it is possible for any peer to coordinate interactions, and therefore to have any blend of control over service orchestration from a highly centralised system to a pure peer-to-peer arrangement, in the simulation a single peer represented the central database and (to keep it even simpler) it generated a set of 10 data elements with quality chosen randomly to be between 0 and 1, so the data elements that were obtained from it by peers tended to have a normal distribution with mean of 0.5. We assumed that the quality that was acceptable to peers was higher than this mean, setting it at 0.8 for all peers, indicating that peers seeking data from the database obtained a mixture of acceptable (mean quality greater than 0.8) and unacceptable results, so the database built up a negative ranking as well as a positive one.

### Experiments on MS/MS protein identification

#### Roles and LCC specifications

**Figure 4:**
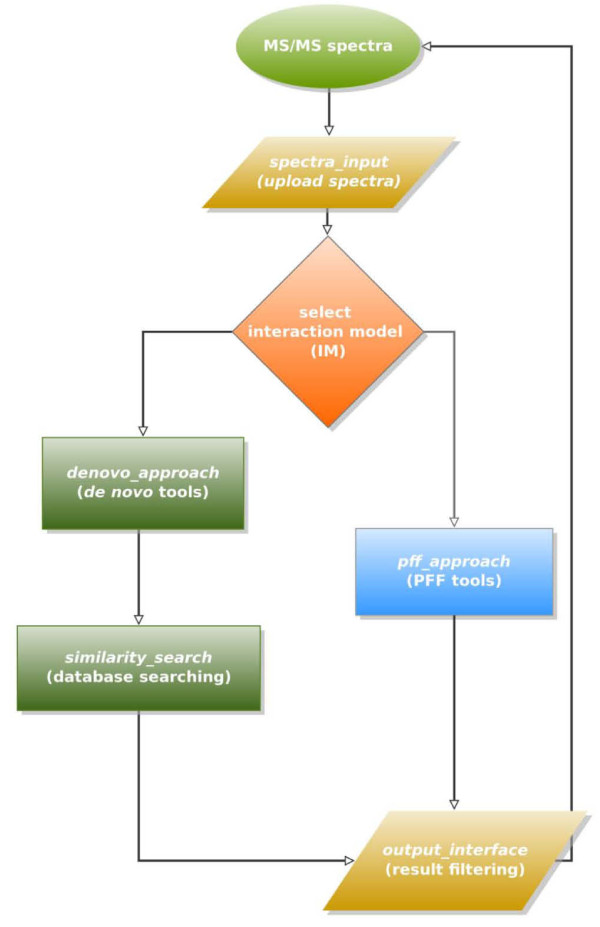
**Peer ranking experiment on MS/MS protein identification**.

Using OpenKnowledge peer-to-peer interaction infrastructure and LCC, we built an experimental environment to access and manipulate multiple web-enabled services or local programs of various types of MS/MS sequencing techniques. These services were configured in parallel pathways (Figure 4) for alternative execution.

As depicted in Figure 4, a peer *spectra_input*, the interaction initiator, uploaded the mass spectrum, and randomly selected an interaction model to execute. If the "*de novo *+ similarity search" interaction model was selected, peers assigned to *denovo_approach *and *similarity_search *were invoked. The analysis result was passed to peer *output_interface *for evaluation. Similarly, if the PFF analysis interaction model was executed, peers responsible for *pff_approach *were invoked. The analysis result was passed to peer *output_interface *for evaluation as well. Evaluation result, success or failure of interaction model, was passed to *spectra_input *in the end.

**Figure 5:**
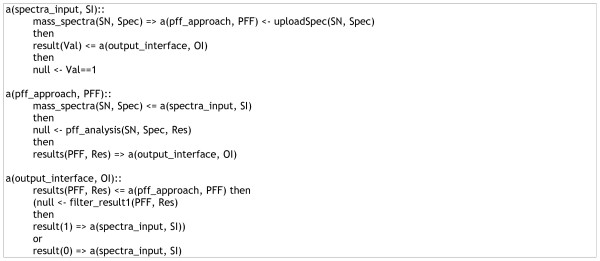
**Interaction pathway for peptide fragement fingerprinting (PFF)**.

According to the PFF interaction pathway as specified in Figure 5, peer *SI *uploaded a mass spectrum (*Spec*) from data source, received the evaluation result (*Val*), and terminated the interaction. Peer PFF received a mass spectrum from *SI*, performed PFF analysis of the spectrum and forwarded the analysis result *Res *to peer *OI *which evaluated the result of PFF analysis and then passed the evaluation result *Val *(0 or 1) to peer *SI*.

**Figure 6:**
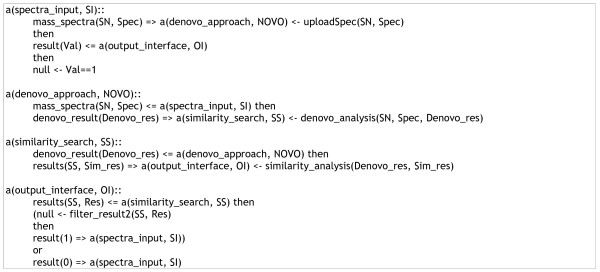
**Interaction pathway for de novo sequencing and database searching**.

Similarly, in the *de novo *interaction pathway shown in Figure 6, peer *SI *uploaded a mass spectrum (*Spec*) from data source, received the evaluation result (*Val*), and terminated the interaction. Peer *NOVO *received a mass spectrum from *SI*, performed *de novo *analysis of the spectrum and forwarded the *de novo *analysis result *Denovo *to peer *SS *for similarity database searching. The peer *OI *received the similarity search result *Res *from *SS*, evaluated the data *Res*, and then forwarded the evaluation result *Val *(0 or 1) to peer *SI*.

Figure 5 specified the LCC specification for the PFF interaction pathway and Figure 6 listed LCC codes for the *de novo *sequencing + database searching pathway. Two roles, *spectra_input *and *output_interface*, were specified in both interaction pathways with the same arguments and interactions. Peers subscribed to the roles of *spectra_input *were responsible for uploading MS/MS spectra and selecting preferred routes for the spectrum interpretation. Peers subscribed to the role *output_interface *were in charge of the re-formating, filtering and displaying of the final result yielded by the interaction model executed. Other roles specified in the two LCC interaction models included *pff_approach*, *denovo_approach*, and *similarity_search*, which performed PFF, *de novo *sequencing, and database searching, respectively.

#### OpenKnowledge components3

OpenKnowledge Components (OKCs) were developed to access and manipulate web servers and/or local programs for MS/MS identification, including algorithms OMSSA and MASCOT subscribed to the role *pff_approach*, PepNovo Win32 Executable [11] and Lutefisk XPv1.0 [12] performed the role *denovo_approach*, and MS-BLAST [13] subscribed to the role *similarity_search*. In this experiment, parameters for MASCOT was set with database *NCBI nr*, enzyme *trypsin*, and all the other parameters were set to default settings. Similarly, OMSSA was run with database *nr*, enzyme *trypsin*, maximum missed cleavages set as "*2*", minimum charge to start using multiply charged products set to "2", all the optional species being selected, and all the other default parameters for ion trap spectrometers. Both Lutefisk and PepNovo were run with their default parameters for doubly charged tryptic peptides on ion trap MS. MS-BLAST was run with default parameters and database *nrdb95*.

OKCs for system roles *spectra_input *and *output_interface *were implemented with GUIs for human users to upload the MS spectra and display MS/MS identification results. The filtering criteria for the results were taken from literature [5,6,14] on different MS/MS identification algorithms as listed in Table 1, to achieve a false discovery rate (FDR) less than 0.1.

**Table 1:** The scores and threshold values for MASCOT, OMSSA, and MS-BLAST.

Peer	Name of score	Threshold	References
MASCOT	MASCOT score	≥ 30	Perkins *et al*., 1999
OMSSA	E-value	≤ 0.1	Geer *et al*., 2004
MS-BLAST	MS-BLAST score	≥ 57	Habermann *et al*., 2004

The results given scores by MASCOT, OMSSA, MS-BLAST were classified to be successes or failures according to their respective thresholds.

#### Experiment execution

The experiment was based on a benchmark dataset with doubly charged tryptic peptides obtained from low-energy ion trap LC/MS/MS runs [11]. Each round went through only one of the possible routes (Figure 4) with one peer to perform the roles specified in the associated LCC codes (Figures 5 and 6).

A single round started with uploading the MS spectra data of peptides to the *spectra_input *peer. In addition to spectrum uploading, the OKC developed for this peer allowed the peer to select the analysis approach, which was either (1) PFF approach or (2) *de novo *sequencing and database similarity searching approach, for MS/MS protein identification. In the cases (e.g., the present study) when none of the approaches was preferred, the system randomly selected one approach. In each route, one of the peers subscribed to the role *pff_approach*, *denovo_approach*, or *similarity_search*, was randomly selected by the system in the experiment. The result of protein identification was sent to the peer performing the role *output_interface *for reformatting and filtering according to the criteria as shown in Table 1. The failure or success message of the execution was finally sent to the peer *spectra_input *in the end of the interaction model. The peer ranking for monitoring the performance of the peers was simply based on counting the numbers of failures and successes.

## Results and discussion

The present study obtained results from experiments on the basic behaviours of simulated peers monitored by PageRank-style peer ranking under OpenKnowledge infrastructure and on the feasibility of combining and ranking real-world services of peptide fragment fingerprinting (PFF) and *de novo *sequencing for MS/MS protein identification.

### PageRank-style ranking of simulated peers

**Figure 7:**
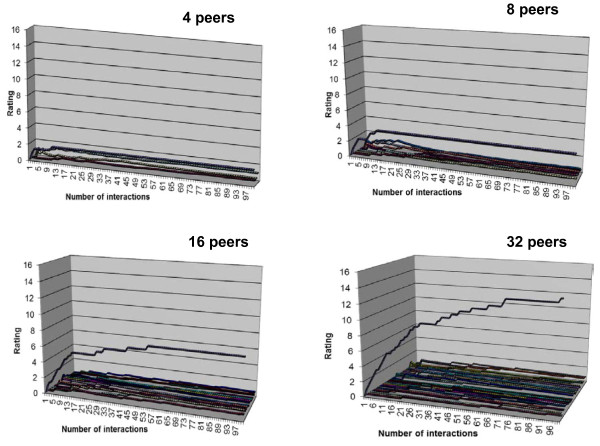
**Power law effect in peer rating with increasing peer numbers**.

In the experiments on simulated peers, PageRank-style ranking under the OpenKnowledge infrastructure worked in a manner similar to ranking in the traditional Web, where a power law effect distinguishes a few dominant pages that obtained high ranks on a topic from the majority that retained lower levels of popularity. This effect encouraged strong competition for popularity and was a key driver of large scale coordination such as the traditional World-wide Web. The tendency to have this effect was observed in the interaction model given (Figure 3) with populations of peers of increasing sizes, including 4, 8, 16, and 32 in the present study. In the experiments each peer had identical behaviour and each peer was compliant with the protocol described in the interaction model, so all interactions run perfectly to completion. The results were depicted in the four graphs of Figure 7. Each line on the graphs was the positive rating of a peer (measured on the y-axis) as it changes while the number of interactions increases (measured on the x-axis). Each graph shows a single peer achieving dominance with a rapid falloff to others with lower rank and a "tail" of low ranking peers. As the number of peers increased this effect became more pronounced and, although separation of dominant peers to the fullest extent takes more interactions with greater peer numbers, the dominant peers began to separate early in all cases.

**Figure 8:**
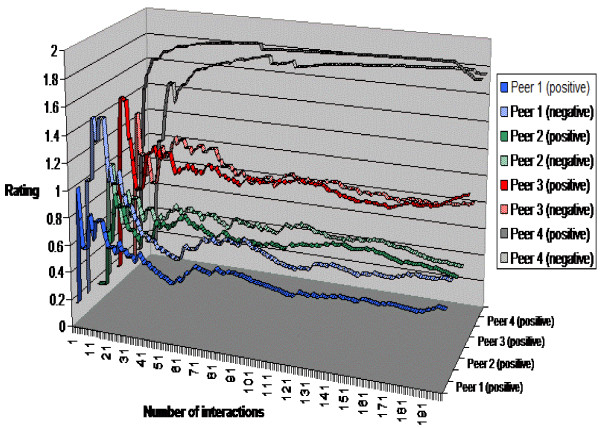
**Peer ranking when peers change their reliability**.

We also investigated the sensitivity of ranking to changes in peer behaviour. For example, whether there is any change in ratings if a peer that initially had been compliant became less compliant in its behaviour. Figure 8 showed the change in positive and negative rating for each of four peers. Instead of having been always compliant, we made peers sometimes non-compliant by making them fail to satisfy the constraint in the interaction model we introduced above, which would broke the protocol. We made the likelihood of failure of a peer increased as the peer had more successful interactions, and decreased as the peer had more failed interactions, so that the likelihood of compliance of each peer alternated over time. The ratings as shown in Figure 8 changed in response to this, as the positive and negative rating lines for each peer tended to twine across each other. As a peer was successful, its positive rating exceeded its negative rating but this success made it more likely to fail to be compliant (because we made each peer decided to behave less compliantly when it had been successful) so its negative rating then climbed and exceeded its positive rating, making it less likely to be selected by other peers. Overlaid on this twining effect was the basic power law effect that we demonstrated in Figure 7, so peer 4 had a dominant rating (both positive and negative, since its popularity attracted many interactions including more that failed as well as more that succeeded), followed by peer 3 and then (some way behind) by peers 1 and 2.

This is an interesting finding as in many data sharing applications we consider the information about the source of the data (e.g., provenance or accuracy) to influence which source we use. Traditionally, provenance was incorporated into data-sharing interactions by devising ontologies for provenance and supplying task-/domain-specific methods for propagating provenance information between peers. We could do this in the OpenKnowledge infrastructure by writing interaction models in LCC that specify provenance propagation along with the message passing needed for "raw" data transmission. This, however, takes engineering effort so it is useful if peer ranking could, alone, take some of the strain of assigning reputation to peers in a data sharing context.

Initially, the central database would become the most popular peer as the other peers had no data so if any of them was asked for data the interaction definitely would fail, increasing their negative ranking while their positive ranking remained unchanged. As the other peers gained data from the database through interaction then those peers lucky enough to get high quality data could then offer it to other peers, raising the positive ranking of the supplier and giving the recipient high quality data with which it could increase its rating.

**Figure 9:**
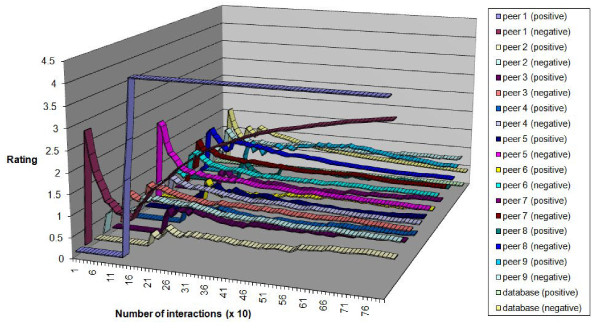
**Ratings for data sharing example**.

Figure 9 shows the behaviour of this system for nine peers interacting with a database. The positive and negative ratings for the top-rated peer (indicated on the diagram) crossed over after about 160 interactions (note that the units on the X axis were in tens of interactions) because the peer acquired a substantial amount of high quality data from the database and was using that to dramatically increase its positive rating. Its high rating made it attractive to other peers which placed demands on it for more data than it possessed so its negative rating climbed too, but not enough to overtake its positive rating.

**Figure 10:**
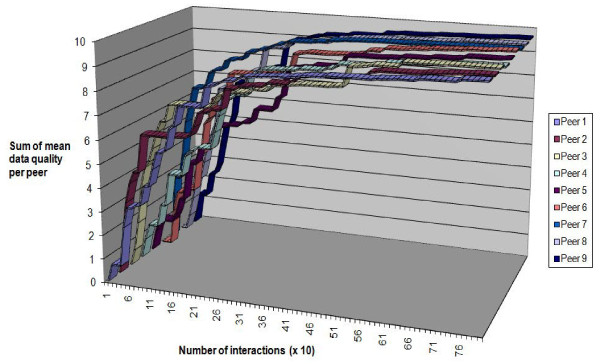
**Ratings for ideal data sharing direct from a single database**.

Figure 10 shows the change in data quality obtained at each peer by taking the sum (for the 10 different queries) of the mean quality of data obtained for that query. The sum would give a possible minimum from 0 (no data obtained) to 10 (all data for all queries at a quality level of 1). After 1000 interactions the sum of mean data quality at each peer rises to approximately 6, which was consistent with the drop in popularity of the central database that we saw in Figure 9. Because peers retained high quality data they were able, once they eventually obtained high quality data from the central database or from another peer, to boost their ratings by contributing to more successful interactions than the central database (which delivers data a more variable quality). The overall effect was to "lift" the data provision from the central database into the peer group.

**Figure 11:**
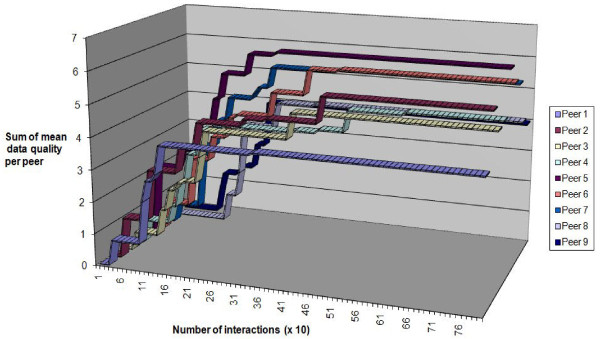
**Ratings for competitive data sharing between peers**.

To show how close our peer to peer method of data sharing comes to the ideal situation, where all the peers can directly poll the database as frequently as they wish, we set up a simulation. In this simulation there was no need to rank peers because every peer interacted only with the database and the database was assumed to have infinite resources so that it could answer every query. Figure 10 shows the change in mean quality of data at each peer under these ideal conditions. As expected, the quality rose quickly to nearly the maximum possible because each peer in this simulation simply polled the database as many times as it took to get the highest quality data. This is ideal but it is impractical when the peer group gets large because the database has to deal with all the interactions and would quickly become overloaded with queries. By contrast, the results of peer to peer sharing (Figure 11) showed a slower improvement in quality but (as seen from Figure 9) this improvement was done by querying across the peer group rather than risking overload on the central database.

### Peer ranking of peers for MS/MS protein identification

Seven peers interacting in the MS/MS protein identification experiment were allowed to interact according to the interaction model (Figures 5 and 6). Each round took a randomly selected MS spectra data (number of spectra used = 239 different spectra) and recorded the ratings and whether the interaction gave a successful protein identification results according to the criteria shown in Table 1. The final ratings were as shown in Table 2. Among all protein identification peers, MS-BLAST performed the best according to the final peer ranking scores. As MS-BLAST processed the protein identification results from two *de novo *sequencing peers, combining *de novo *sequencing peers (e.g., PepNovo and Lutefisk) would improve the final ratings. Due to the consistently good performance of MASCOT, it was selected more frequently than OMSSA and thus MASCOT outperformed OMSSA.

**Table 2:** Peer ranking in MS/MS protein identification experiment.

Rank	Peer	Peer ranking score	Total runs	Number of successful runs	Number of failed runs
1	output_interface and spectra_input	0.308	239	110	129
2	MS-BLAST	0.162	130	47	83
3	MASCOT	0.122	68	53	15
4	PepNovo	0.104	61	30	31
5	Lutefisk	0.059	69	17	52
6	OMSSA	0.023	41	10	31

The interaction with seven peers involved in the PFF and *de novo *approaches was executed 238 times. The ranking of the peers was based on the number of times executed and the numbers of successful and failed interactions.

PFF algorithm MASCOT was rated better in peer ranking than the two *de novo *sequencing tools. This result was consistent to the previous finding that PFF analysis was more accurate than *de novo *sequencing [12]. For *de novo *sequencing approach, the ratings of PepNovo were higher than that of Lutefisk. Even though this experiment did not evaluate how accurate was each round of protein identification for each sequence output, the peer ranking results in this experiment were consistent with the performance evaluation result of different *de novo *sequencing programs by Frank and Pevzner [11].

**Figure 12:**
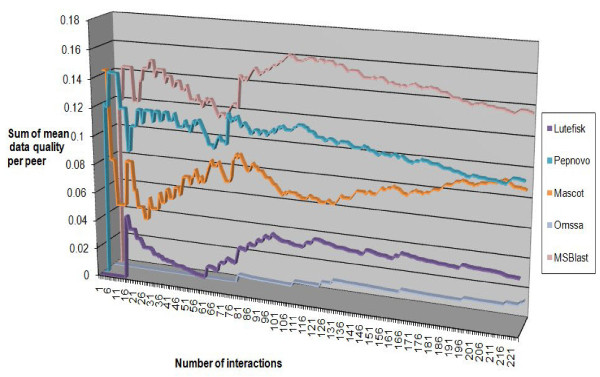
**The changes of peer ranking scores during interactions**.

There was a directed change of ratings during the whole process of interaction as shown in Figure 12. The ratings became relatively stable after 100 rounds of interaction. The differences among peers became obvious after 25 rounds of interaction. The changes in ratings followed the behavioural patterns of peer ranking as demonstrated in the experiments using simulated peers.

As seen in the experiments of the present study, peer ranking is applicable to monitoring peer-to-peer interactions under the OpenKnowledge infrastructure. With more OKCs built for more practical tasks (not limited to protein identification) and various performance feedback mechanisms like peer ranking, we would be able to include more peers for large-scale experimentation on various other issues such as trust [15].

## Conclusion

This study demonstrated a use of peer ranking to support automated experimentation in open and peer-to-peer environments. Both simulated and real-world experiments in the present study showed that the OpenKnowledge infrastructure with peer ranking capability can serve as an evaluative environment for automated experimentation.

## Competing interests

The authors declare that they have no competing interests.

## Authors' contributions

DR designed this study and run the experiments on simulated peers. SL proposed the experimentation on MS/MS protein identification. XQ and SL selected MS/MS protein identification services for the experiments. PB and XQ wrote the LCC specifications for protein identification. QL implemented the OKCs and MS/MS peer ranking. XQ and QL run the protein identification experiments. All authors (DR, SL, XQ, PB, QL, MC, and DG) contributed ideas of the current implementation of peer ranking under OpenKnowledge infrastructure. DR, SL, and XQ drafted the manuscripts. DR and SL revised the manuscripts in accordance with the reviewers' comments. All authors read and approved the final manuscript.
